# Supplementary material for: Water nanolayer facilitated solitary-wave-like blisters in MoS2 thin films
Source: Nat Commun. 2023 Jul 19;14:4324. doi: 10.1038/s41467-023-40020-7 (PMC10356837; doi:10.1038/s41467-023-40020-7)
Supplement: Supplementary file 1 — Supplementary Information [file 41467_2023_40020_MOESM1_ESM.pdf]

Supplementary Information

Water nanolayer facilitated solitary-wave-like blisters in MoS_2 thin films

Enze Wang[1#], Zixin Xiong[2#], Zekun Chen[2], Zeqin Xin[1], Huachun Ma[2], Hongtao Ren[3], Bolun Wang[1], Jing Guo[1], Yufei Sun[1], Xuewen Wang[1], Chenyu Li[1], Xiaoyan Li[2*] and Kai Liu[1*]

1. Characterization of MoS_2 films

2. Experimental equipment

3. Typical morphology of arc buckles with large radii

4. Propagation speed of the SWLB

5. Relationship between δ and b

6. Heights of SWLB and web buckles

7. Calculation of interfacial adhesion and bonding strength

8. Modulus and hardness measured by nanoindentation tests

9. Calculation of the surface energy

10. DFT calculations of diffusion of water molecules at the interface

11. Measurement of water nanolayer thickness by atomic force microscopy

12. Estimation of the capillary force of the interfacial water layer

13. Bubbles formed in the interfacial water nanolayer

14. Theoretical model for SWLB propagation

15. Prediction of the equilibrium positions by our theoretical model

1. Characterization of MoS_2 films

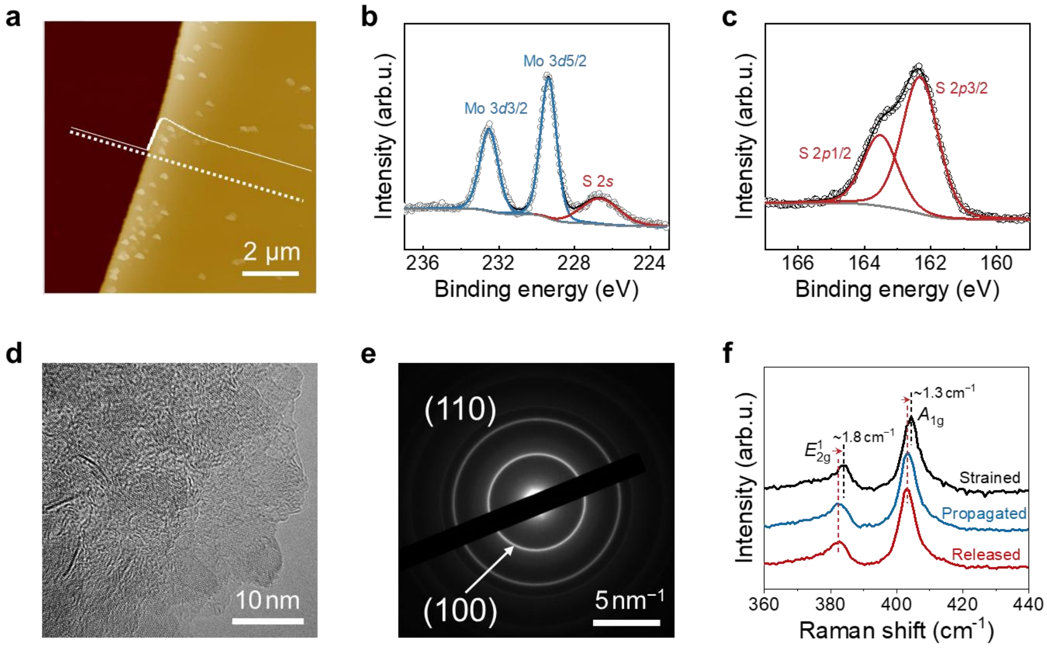

Supplementary Fig. 1 | Characterization of MoS_2 thin films. a, AFM image of a MoS_2 film with a thickness of ~320 nm**. b–c**, XPS Mo 3*d* (**b**) and S 2*p* (**c**) spectra of a MoS_2 film, respectively. **d**, HRTEM image of the MoS_2 film. **e**, SAED pattern of the MoS_2 film. **f**, Raman spectra of an as-grown flat MoS_2 film area (strained), the same area where the SWLB propagated (propagated), and the same area where strain was fully released after peeled off (released). Source data are provided as a Source Data file.

The X-ray photoelectron spectrum (XPS) of the as-prepared films shows two peaks at 229.4 eV and 232.5 eV corresponding to Mo 3*d*5/2 and 3*d*3/2 of MoS_2, and doublets at 162.3 eV and 163.5 eV ascribed to S 2*p*3/2 and 2*p*1/2[1, 2] (Supplementary Fig. 1b–c). The high-resolution transmission electron microscope (HRTEM) image and selected area electron diffraction (SAED) pattern reveal that the MoS_2 films consist of mainly polycrystalline phases and a few amorphous phases (Supplementary Fig. 1d–e). The Raman spectrum collected from the strained film shows that the in-plane vibrational mode E^1_{2g} of MoS_2 blueshifts by ~1.8 cm^{-1} compared to that collected from the released MoS_2 film, where the stress should be fully relaxed. And the Raman spectrum collected from the film after SWLB propagated well match those of the same area peeled off, which indicates that the strain in the MoS_2 film is completely

released after the SWLB propagated (Supplementary Fig. 1f). This blueshift of E_{2g}^{1} suggests that the as-prepared MoS_2 film bears a biaxial compressive strain of ~0.35% at the film-substrate interface[3], which may result from the mismatch of thermal expansion between MoS_2 and sapphire during growth.

2. Experimental equipment

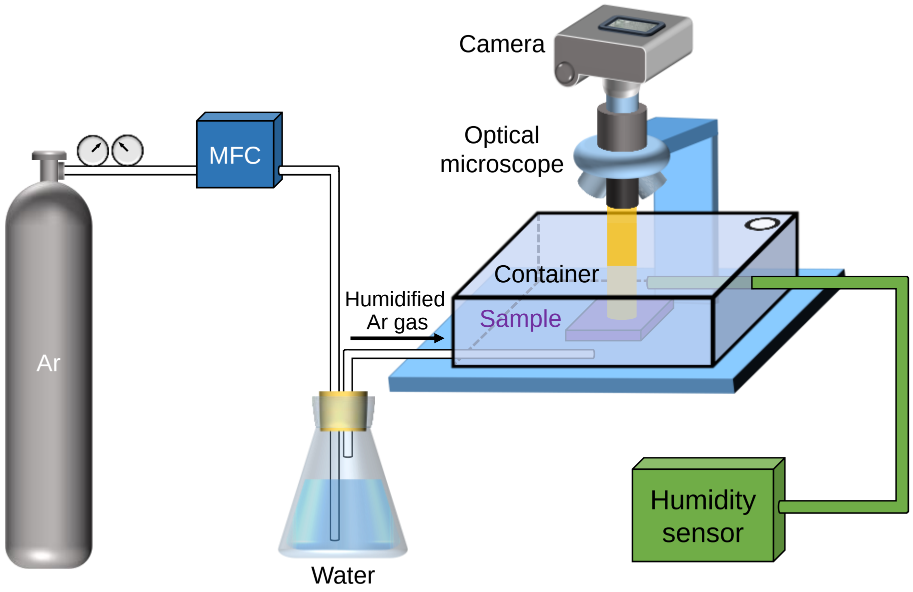

Supplementary Fig. 2 | Schematic illustration of the homemade humidity control equipment. The equipment includes an Ar gas cylinder, a mass flow meter, a conical flask, a container, and a humidity sensor.

3. Typical morphology of arc buckles with large radii

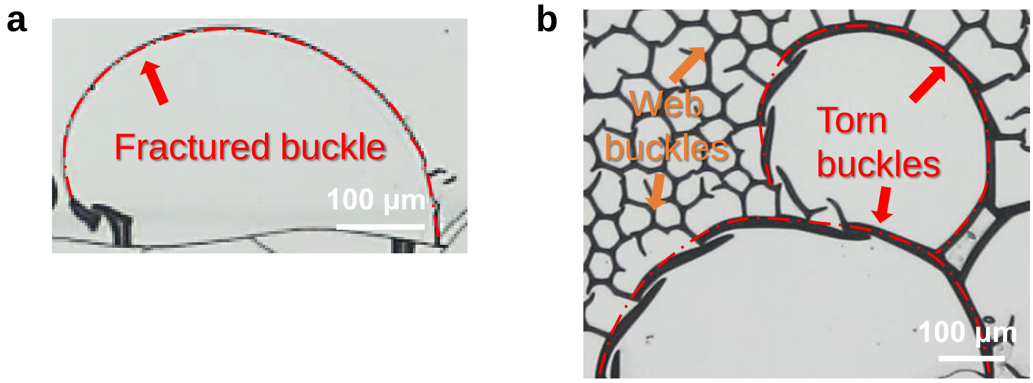

Supplementary Fig. 3 | Typical morphology of arc buckles with large radii. **a**, Fractured buckle with a circular crack. **b**, Torn buckles marked by red lines and web buckles rooted from arc buckles.

During propagation, the increasing curvature radius of the arc buckles leads to a larger bending energy, which may eventually tear themselves. For arc buckles with large radii, two types of morphology are often seen. One is a circular crack originating from the central line of the arc buckles (Supplementary Fig. 3a), and the other is some small buckles formed by the breakage of the large buckles (Supplementary Fig. 3b). In Fig. 2i, both circular cracks (fractured buckles) and torn small buckles are included in the broken buckles.

4. Propagation speed of the SWLB

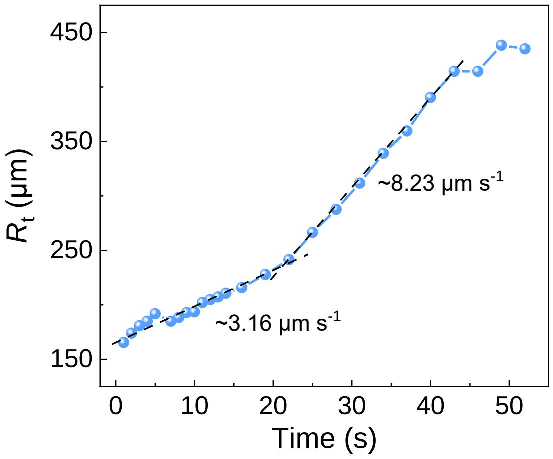

Supplementary Fig. 4 | R_t of an arc buckle as a function of time. Source data are provided as a Source Data file.

The increase in R_t follows an almost linear relationship with time. Thus, the propagation speeds of the arc buckles are obtained by fitting the slope of R_t versus time curve and fall in the range of several micrometers per second.

5. Relationship between δ and b

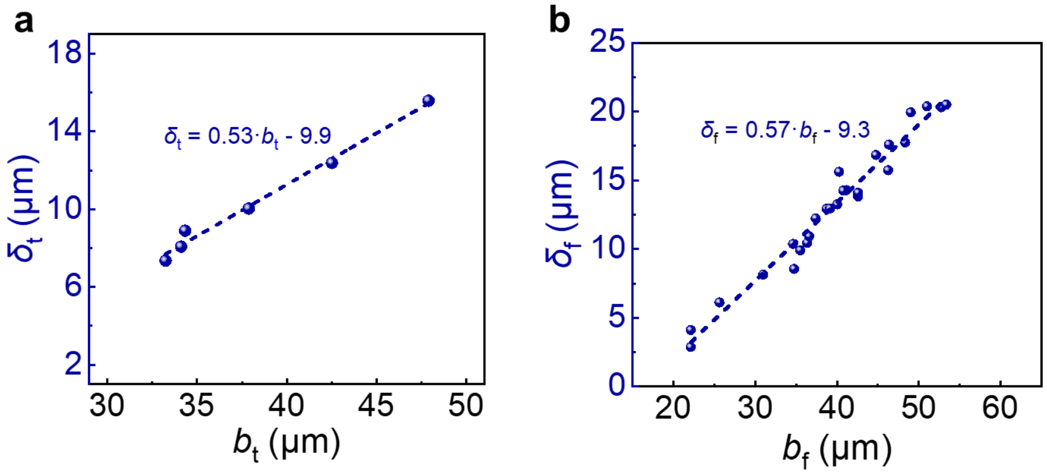

Supplementary Fig. 5 | Linear relationship between δ and b of the arc buckles. a, Transient height δ_t as a function of b_t for the arc buckle in Fig. 2 a–f. **b**, Final height δ_f as a function of b_f measured from some arc buckles. Source data are provided as a Source Data file.

Except for the linear relationship between δ_t and b_t, the final height δ_f also obeys a linear relationship with b_f, as shown in Supplementary Fig. 5. Based on the theoretical model of straight-sided buckles[4], the relation between δ and b can be described by the following equation:

$$\sigma_c = \frac{\pi^2 E_f}{12(1-\nu_f^2)}\left(\frac{t}{b}\right)^2 \tag{S1}$$

$$\sigma_r = \gamma_\sigma \sigma_c \left[\frac{3}{4}\left(\frac{\delta}{\gamma_\delta t}\right)^2 + 1\right] \tag{S2}$$

$$\frac{\delta}{t} = \gamma_\delta \sqrt{\frac{4}{3}\left(\frac{\sigma_r}{\gamma_\sigma \cdot \sigma_c} - 1\right)} = \gamma_\delta \sqrt{\frac{4}{3}\left(\frac{12(\varepsilon_{11} + \nu_f \varepsilon_{22})}{\pi^2 \gamma_\sigma}\left(\frac{b}{t}\right)^2 - 1\right)} \sim \frac{b}{t} \tag{S3}$$

where E_f, ν_f, and t are the modulus, Poisson's ratio, and thickness of the film, respectively; σ_c is the critical stress for the buckling mode with half width b; σ_r is the residual stress; γ_δ and γ_σ are two parameters related to the properties of the film and the substrate, respectively; and ε_{11} and ε_{22} are the residual strains in the film.

As evidenced by the above equations, the relationship between δ and b is almost linear, and the fitting slope is related to the residual strain in the film. Since the width of the SWLB increases gradually during its propagation, the buckling height also enlarges and maintains this linear relationship.

6. Heights of SWLB and web buckles

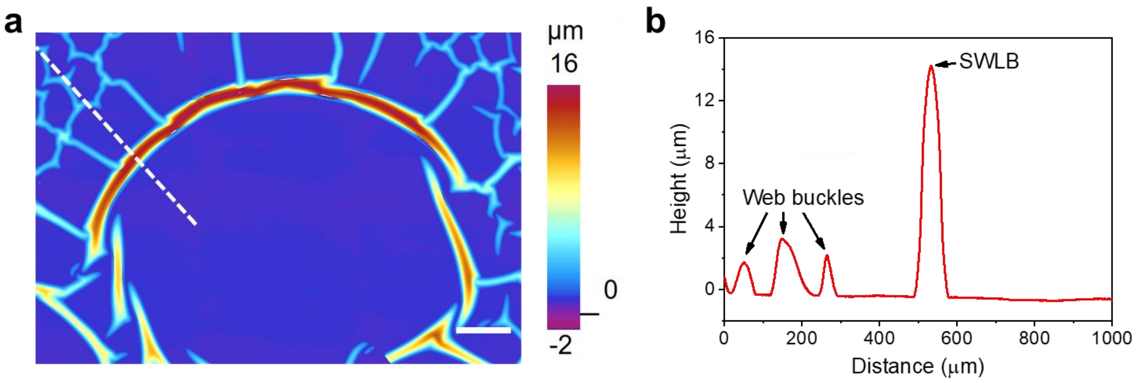

Supplementary Fig. 6 | Heights of different blisters. (a) Surface morphology of a SWLB and some adjacent web buckles characterized by a laser confocal microscope. Scale bar, 200 μm. (b) Height profile along the white line shown in (a). Source data are provided as a Source Data file.

The surface morphology of SWLB is measured from the SWLB and web buckles in Fig. 2f along the white line. The heights of web buckles are 2, 2.5 and 3.5 μm, respectively, and the height of SWLB is ~14 μm, revealing that the height of SWLB is far beyond the web buckle amplitude.

7. Calculation of interfacial adhesion and bonding strength

Supplementary Fig. 7 shows a schematic illustration of the in situ measurement of the interfacial adhesion and bonding strength. The adhesions are derived from the traction-displacement curve (Fig. 3a). The adhesion forces under 60% and 80% RH are 13.9 N and 5.0 N, respectively. By measuring the area of the film that is peeled from the substrate, the bonding strength can be determined. These results are listed in Supplementary Table 1.

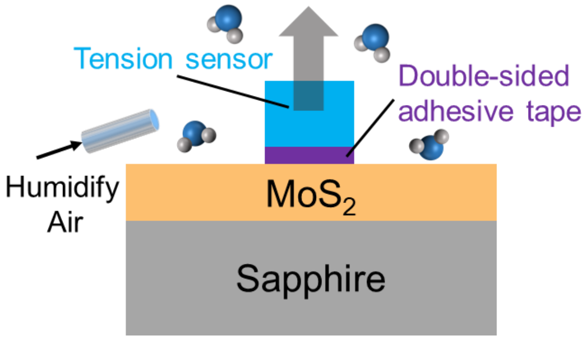

Supplementary Fig. 7 | Schematic illustration of the in situ measurement of interfacial adhesion and bonding strength.

Supplementary Table 1: Adhesion, peeled area, and bonding strength at different humidities

Relative humidity	Adhesion	Peeled area	Bonding strength
~60%	13.9 N	17.9 mm^2	0.78 MPa
~80%	5.0 N	21.1 mm^2	0.24 MPa

8. Modulus and hardness measured by nanoindentation tests

The elastic modulus and hardness of the films were measured by a nanoindentation system (Keysight G200) using the continuous stiffness method[5]. The modulus of the film is extracted from the apparent modulus by the J. Hay model[6] to exclude substrate effects (Supplementary Fig. 8). The data at small indentations suffer from more uncertainty due to the wear of the Berkovich indenter; therefore, an indentation depth from 30% to 50% of the thickness is used for estimating modulus and hardness. The film thickness is ~350 nm, so the indentation depth used for calculation ranges from 105 to 175 nm, and the results are listed in Supplementary Table 2.

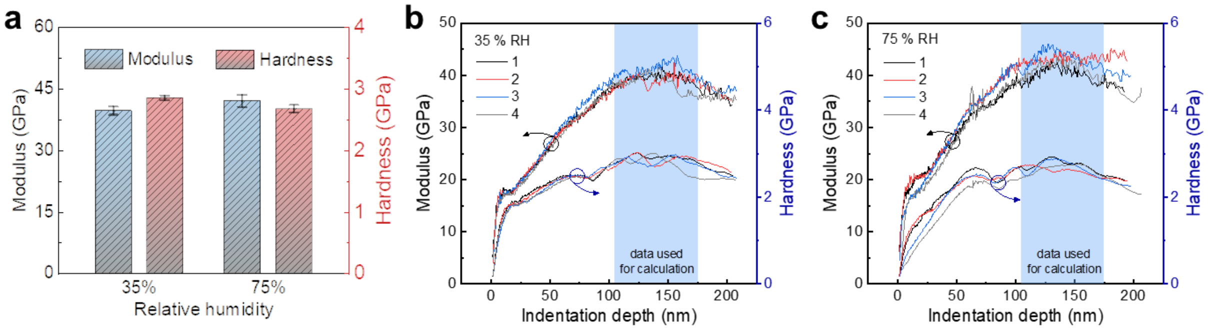

Supplementary Fig. 8 | Modulus and hardness of the MoS_2 film (**a**) derived from the continuous stiffness method by nanoindentation at 35% RH (**b**) and 75% RH (**c**). The data in (**a**) are collected from the blue regions of (**b**) and (**c**), and the error bars represent standard deviations from four measurements of modulus or hardness values at each humidity. Source data are provided as a Source Data file.

Supplementary Table 2: Modulus and hardness of the film at different humidities

Relative humidity	Position	Modulus (GPa)	Hardness (GPa)
35%	1	39.4	2.90
	2	39.1	2.86
	3	41.2	2.83
	4	39.0	2.82
75%	1	40.2	2.75
	2	43.1	2.62
	3	43.4	2.69
	4	41.4	2.64

9. Calculation of the surface energy

The surface energy of the MoS_2 film and sapphire substrate is calculated based on the OWRK method[7, 8]. Young's equation gives

$$\gamma_{sv} = \gamma_{sl} + \gamma_{lv}\cos\theta \tag{S4}$$

where θ is the contact angle and γ_{sv}, γ_{sl} and γ_{lv} are the solid surface energy, solid/liquid interfacial energy, and liquid surface tension, respectively.

The OWRK method divides the solid surface energy into two components: the polar component and the dispersive component. The interfacial energy γ_{sl} is interpreted as the geometric mean of the disperse component and the polar component of the surface tension or surface free energy:

$$\gamma_{sl} = \gamma_{sv} + \gamma_{lv} - 2\left(\sqrt{\gamma_{sv}^{D}\gamma_{lv}^{D}} + \sqrt{\gamma_{sv}^{P}\gamma_{lv}^{P}}\right) \tag{S5}$$

where γ_{sv}^{D} and γ_{lv}^{D} are the dispersive components of the solid and liquid surface energies, while γ_{sv}^{P} and γ_{lv}^{P} are the polar components.

Substituting Eq. (S5) into Eq. (S4), the OWRK equation will be given,

$$\gamma_{lv}\left(1+\cos\theta\right) = 2\left(\sqrt{\gamma_{sv}^{D}\gamma_{lv}^{D}} + \sqrt{\gamma_{sv}^{P}\gamma_{lv}^{P}}\right) \tag{S6}$$

To determine the dispersive component γ_{sv}^{D} and polar component γ_{sv}^{P} of the solid surface energy, two kinds of liquid with known γ_{lv}^{D} and γ_{lv}^{P} but different polarities are required to measure the contact angles on the surface of the tested solid.

In our experiment, water and diiodomethane are chosen as the testing liquids, and the corresponding contact angles of these two liquids on the MoS_2 film and sapphire substrate are shown in Supplementary Fig. 9. The dispersive and polar components of the liquid surface tension are listed in Supplementary Table 3, and the calculated surface energy and adhesion work of MoS_2 and sapphire are listed in Supplementary Table 4. The adhesion work is calculated based on the following equation:

$$W_{\text{material-water}} = \gamma_{\text{material-vapor}} + \gamma_{\text{water-vapor}} - \gamma_{\text{material-water}} \tag{S7}$$

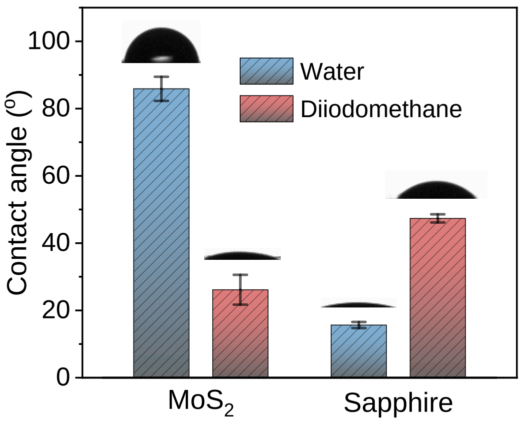

Supplementary Fig. 9 | Contact angles of water and diiodomethane on MoS_2 film and sapphire substrate. Error bars represent the standard errors, each of which is extracted from three independent tests. Source data are provided as a Source Data file.

Supplementary Table 3: Dispersive and polar components of the surface tension for testing liquids.

Liquid	γ_{lv} (mN m^{-1})	γ_{lv}^{D} (mN m^{-1})	γ_{lv}^{P} (mN m^{-1})
Water	72.1	19.9	52.2
Diiodomethane	50.0	47.4	2.6

Supplementary Table 4: The calculated results for the two tested materials.

Material	γ_{sv} (mN m^{-1})	γ_{sv}^{D} (mN m^{-1})	γ_{sv}^{P} (mN m^{-1})	$W_{material\text{-}water}$ (mJ m^{-2})
MoS_2	45.1	43.5	1.6	77.3
Sapphire	69.5	19.7	49.8	141.5

The adhesion work between MoS_2 and water is 77.3 mJ m^{-2}, indicating that the replacement of the MoS_2/air interface by the MoS_2/water interface is energetically favorable. This is a microscopic origin of the re-adhesion phenomenon at the tail side of the arc buckles.

10. DFT calculations of diffusion of water molecules at the interface

To more clearly show the possibility of the diffusion of water molecules at the interface, we performed density functional theory (DFT) calculations via the Vienna Ab initio Simulation Package (VASP) program.[9] The generalized gradient approximation with the Perdew–Burke–Ernzerhof type exchange-correlation functional (GGA-PBE) and projector augmented wave (PAW) method were adopted in all the calculations.[10, 11] The plane-wave energy cutoff was fixed at 530 eV, and a 3 × 3 × 1 Monkhorst–Pack grid was used for the k-point sampling. The van der Waals interactions were involved by using the DFT-D3 method of Grimme.[12] A smearing of 0.1 eV was used. The convergence of energy and maximum force were set to 10^{-5} eV and 5×10^{-3} eV Å^{-1}, respectively. For the calculations of the adsorption energies of H_2O molecules inserted into the interface of MoS_2/sapphire, a (2 × 2) supercell of sapphire (001) with three atomic layers was built. Then, a (3 × 3) unit cell of MoS_2 was adsorbed on the sapphire surface, with the contact distance *d* between MoS_2 and sapphire set to 3, 4, 5, 6, and 7 Å. An H_2O molecule was inserted between MoS_2 and sapphire.

The adsorption energy of the H_2O molecule is defined as

$$E_a = E_{MoS_2/H_2O/sapphire} - E_{MoS_2/sapphire} - E_{H_2O}$$

where $E_{MoS_2/H_2O/sapphire}$ and $E_{MoS_2/sapphire}$ are the energies of the MoS_2/sapphire model with and without the H_2O molecule, respectively. E_{H_2O} is the energy of the H_2O molecule in vacuum.

As shown in Supplementary Fig. 10, the adsorption energy is positive at the contact distance of 3 Å. As the van der Waals gap of CVD-grown MoS_2 on sapphire is usually about 3 Å,[13] this calculation result shows that water molecules could hardly diffuse into the film-substrate interface for perfect MoS_2 films. However, the physical gap between MoS_2 and sapphire at some local edges or defects would be larger than the van der Waals gap. When the physical gap is larger than 3 nm, the adsorption energy of the H_2O molecule rapidly decreases to negative as the contact distance *d* increases up to 7 Å, which means that water molecules could more easily infiltrate into the interface and further expand the physical gap. The expansion of physical gap further supports a spontaneous infiltration of water molecules and formation of water nanolayer.

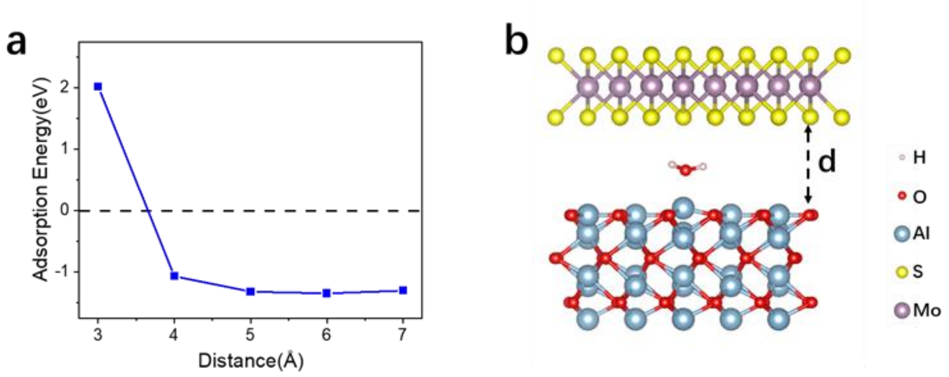

Supplementary Fig. 10 | Calculated adsorption energies of a water molecule between MoS_2 and sapphire. (a) Adsorption energies of a water molecule with different contact distances *d* ranging from 3 to 7 Å. (b) Side view of MoS_2/H_2O/sapphire configuration. Source data are provided as a Source Data file.

11. Measurement of water nanolayer thickness by atomic force microscopy

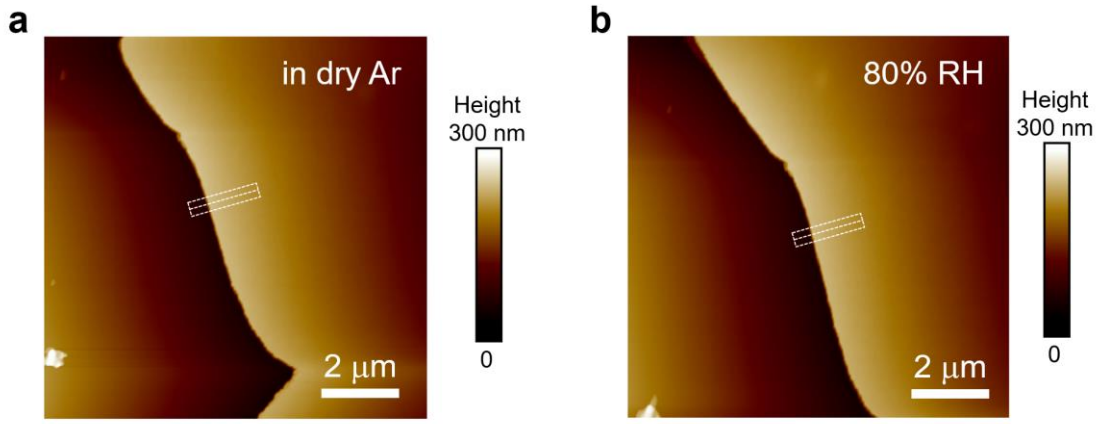

Supplementary Fig. 11 | Atomic force microscope (AFM) height images of a MoS_2 film in dry Ar (**a**) and at 80% RH (**b**) at room temperature.

During the characterization, the AFM (Bruker Multimode 8) chamber was purged with 100 sccm dry Ar (RH=0) gas for 1 h, and then the sample was scanned under peak-force mode. After that, the Ar gas was humidified by bubbling through deionized water in a conical flask, which maintained an RH of 80% for 1 h, and the sample was scanned by AFM again. The height profile lines were extracted from AFM images at 5 different points for each MoS_2 film. The increase in the sample thickness reflects the thickness of the water nanolayer emerging at the film-substrate interface at high humidity, as shown in Fig. 3c.

12. Estimation of the capillary force of the interfacial water layer

According to the Laplace equation, the meniscus water between two surfaces will create an extra pressure as follows:

$$\Delta p = \gamma_{H_2O}\left(\frac{1}{R_1}+\frac{1}{R_2}\right) \tag{S11}$$

where Δp is the Laplace pressure, γ_{H_2O} is the surface tension of water, and R_1 and R_2 are the two principle curvature radii of meniscus water.

At equilibrium, the meniscus curvature is related to the relative vapor pressure described by the Kelvin equation,

$$\left(\frac{1}{R_1}+\frac{1}{R_2}\right)^{-1} = r_k = \frac{\gamma_{H_2O}V_m}{RT\log(p/p^{sat})} \tag{S12}$$

where r_k is the Kelvin radius of the water, V_m is the mole volume, R is the ideal gas constant, T is the Kelvin temperature, and p and p^{sat} are the actual and saturated pressures, respectively. For water at 20°C, $\frac{\gamma_{H_2O}V_m}{RT} \approx 0.54\ \text{nm}$.

In a humid environment, two adjacent hydrophilic surfaces will undergo capillary condensation, and the critical separation $d_{0,cap}$ is given by

$$d_{0,cap} = R_1\left(\cos\theta_{MoS_2}+\cos\theta_{Sapphire}\right) \approx r_k\left(\cos\theta_{MoS_2}+\cos\theta_{Sapphire}\right) \tag{S13}$$

where θ_{MoS_2} and $\theta_{sapphire}$ are the contact angles of the MoS_2 film and sapphire substrate with water, respectively.

Assuming the volume of the condensate water is constant in the vicinity of the buckles, one has $V = A_{0,cap}d_{0,cap}$, where V is the volume of the condensate water, and $A_{0,cap}$ is the wetted surface area of the pristine condensate water.

For meniscus water with a thickness of d, the capillary force per unit area can be derived as follows[14]:

$$F_{\mathrm{cap}} = \Delta p \frac{A}{A_{0,\mathrm{cap}}} = \Delta p \frac{d_{0,\mathrm{cap}}}{d} = \frac{\gamma_{\mathrm{H_2O}}}{R_1} \frac{r_{\mathrm{k}}(\cos\theta_{\mathrm{MoS_2}} + \cos\theta_{\mathrm{Sapphire}})}{d}$$
$$= \frac{\gamma_{\mathrm{H_2O}}}{d/(\cos\theta_{\mathrm{MoS_2}} + \cos\theta_{\mathrm{Sapphire}})} \frac{r_{\mathrm{k}}(\cos\theta_{\mathrm{MoS_2}} + \cos\theta_{\mathrm{Sapphire}})}{d} \quad \text{(S14)}$$
$$= \frac{\gamma_{\mathrm{H_2O}} r_{\mathrm{k}}(\cos\theta_{\mathrm{MoS_2}} + \cos\theta_{\mathrm{Sapphire}})^2}{d^2}$$

where F_{cap} is the capillary force per unit area, A is the actual wetted area, and d is the thickness of the interfacial water layer.

For 80% RH, $r_{\mathrm{k}} \approx -2.42$ nm, considering $\theta_{\mathrm{MoS_2}} \approx 86°$, $\theta_{\mathrm{sapphire}} \approx 16°$ and $\gamma_{\mathrm{H_2O}} = 72.1$ mN m^{-1}, the F_{cap} of the interfacial water layer with a thickness of 2 nm (4 nm) is ~46.4 MPa (~11.6 MPa).

13. Bubbles formed in the interfacial water nanolayer

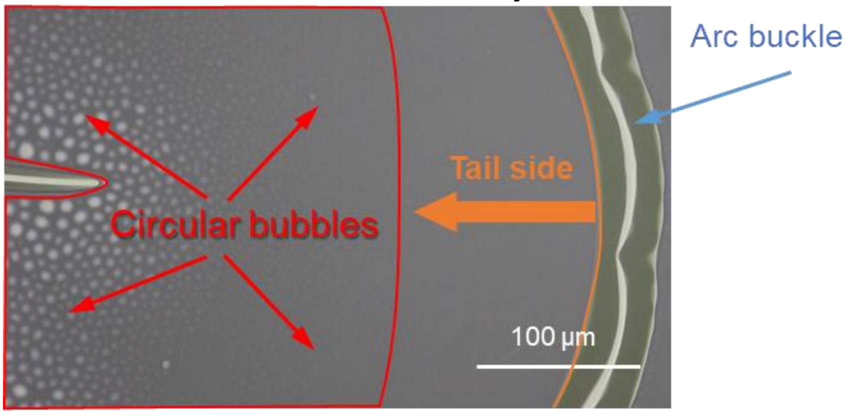

Supplementary Fig. 12 | Circular bubbles formed in the interfacial water nanolayer at the tail side of arc buckles observed from the side of the double-polished sapphire substrate. These formed bubbles indicate that the capillary force in the interfacial water nanolayer has reached its upper limit.

14. Theoretical model for SWLB propagation

We developed a theoretical model to complement the experiments. In this model, the arc buckles are simplified to be axisymmetric, that is, the buckling deformation along the circumferential direction is uniform. The film can be divided into three areas: the inner flat area swept by an arc buckle (marked as S1 in Fig. 4d), the buckling area where the film buckles and delaminates from the substrate (S2), and the outer area where the film is pristine and the residual stress is not released (S3). It is noted that the inner area is bonded to the substrate due

to the capillary effect of the interfacial water nanolayer, but the residual stress in this area has been fully released.

According to the buckling morphologies (Fig. 2g) observed experimentally, the out-of-plane displacement w of the buckling area is assumed to be

$$w = \frac{\delta}{2}\left\{1+\cos\left[\frac{\pi}{b}\left(r-r_0\right)\right]\right\} \tag{S15}$$

where r is the polar coordinate, r_0 is the position of the arc buckle along the polar axis, and $2b$ and δ are the width and height of the arc buckle, respectively. Such a cosine function can accurately capture the sectional profiles of the buckling film from our experiments (Supplementary Fig. 13).

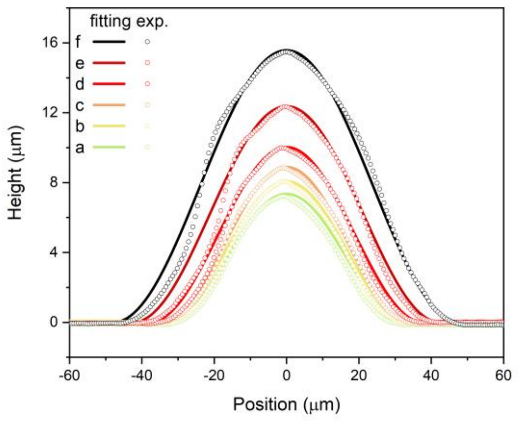

Supplementary Fig. 13 | Section profiles fitted by cosine functions. The solid lines correspond to the cosine fitting of experimental height-profile data (circular hollow dots) crossing the middle of arc buckles marked by white lines in Fig. 2a–f, from the inside out. Source data are provided as a Source Data file.

In the Cartesian coordinate system (x_1, x_2), the out-of-plane deflection w of the buckling film can be described by the following Föppl–von Kármán equation:

$$D\nabla^4 w + \left(N_{11}w_{,11} + N_{22}w_{,22} + 2N_{12}w_{,12}\right) = -p_{\mathrm{S}} \tag{S16}$$

$$\frac{1}{E_{\mathrm{f}}t}\nabla^4 F = {w_{,12}}^2 - w_{,11}w_{,22} \tag{S17}$$

where ∇ is the nabla differential operator, "," represents the differentiation,

$D = E_f t^3 / \left[12\left(1-\nu_f^2\right)\right]$ represents the bending stiffness of the film, E_f, ν_f, and t are the elastic modulus, Poisson's ratio and the thickness of the film, respectively, F is the Airy stress function, p_S is the normal force acting on the film by the substrate, and N represents the tension in the film, and its components include $N_{11}=F,_{22}$, $N_{22}=F,_{11}$, and $N_{12}=-F,_{12}$. For the axisymmetric problem, Eq. (S17) can be simplified as

$$r\frac{\mathrm{d}}{\mathrm{d}r}\left[\frac{1}{r}\cdot\frac{\mathrm{d}}{\mathrm{d}r}\left(r^2\cdot N_r\right)\right]+\frac{E_f t}{2}\left(\frac{\mathrm{d}w}{\mathrm{d}r}\right)^2=0 \tag{S18}$$

The tension along the radial direction can be obtained by solving Eq. (S18):

$$N_r=-\frac{E_f t}{2}\frac{1}{r^2}\int r\left(\int\frac{1}{r}\left(\frac{\mathrm{d}w}{\mathrm{d}r}\right)^2\mathrm{d}r\right)\mathrm{d}r \tag{S19}$$

According to the strain continuity, the boundary conditions of the delaminated area are given by

$$\begin{aligned}\varepsilon_\theta\big|_{r=r_1}&=0\\ \varepsilon_\theta\big|_{r=r_2}&=-\varepsilon_0\end{aligned} \tag{S20}$$

where $r_1 = r_0 - b$ and $r_2 = r_0 + b$ are the inner and outer boundaries of the delaminated area, respectively, and ε_0 is the residual strain in the nondelaminated film. Furthermore, the deformation compatibility condition is expressed as

$$\int_{r_1}^{r_2}\varepsilon_r\mathrm{d}r=-\varepsilon_0\left(r_2-r_1\right)+\int_{r_1}^{r_2}\frac{w,_r{}^2}{2}\mathrm{d}r \tag{S21}$$

During deformation, the total energy of the buckled film includes the bending energy U_b and the in-plane stretching energy U_s. These two energies are written as

$$\begin{aligned}U_b&=\int_{S_2}\frac{D}{2}\left[\left(\frac{\partial^2 w}{\partial x^2}+\frac{\partial^2 w}{\partial y^2}\right)^2+2\left(1-\nu_f\right)\left(\left(\frac{\partial^2 w}{\partial x\partial y}\right)^2-\frac{\partial^2 w}{\partial x^2}\frac{\partial^2 w}{\partial y^2}\right)\right]\mathrm{d}S\\ &=\int_{S_2}\frac{D}{2}\left[\left(\frac{\mathrm{d}^2 w}{\mathrm{d}r^2}\right)^2+\frac{1}{r^2}\left(\frac{\mathrm{d}w}{\mathrm{d}r}\right)^2+\frac{2\nu_f}{r}\frac{\mathrm{d}^2 w}{\mathrm{d}r^2}\frac{\mathrm{d}w}{\mathrm{d}r}\right]\mathrm{d}S\end{aligned} \tag{S22}$$

$$\begin{aligned}U_s&=\int_{S_2}\frac{1}{2E_f t}\left[\left(\frac{\partial^2 F}{\partial x^2}+\frac{\partial^2 F}{\partial y^2}\right)^2+2\left(1+\nu_f\right)\left(\left(\frac{\partial^2 F}{\partial x\partial y}\right)^2-\frac{\partial^2 F}{\partial x^2}\frac{\partial^2 F}{\partial y^2}\right)\right]\mathrm{d}S\\ &=\int_{S_2}\frac{1}{2E_f t}\left[\left(\frac{\mathrm{d}N_r}{\mathrm{d}r}r+N_r\right)^2+N_r{}^2-2\nu_f N_r\left(\frac{\mathrm{d}N_r}{\mathrm{d}r}r+N_r\right)\right]\mathrm{d}S\end{aligned} \tag{S23}$$

Thus, the total energy U_t of the film in the inner area and buckled area can be expressed as

$$U_{\mathrm{t}} = S_2\left(U_{\mathrm{b}} + U_{\mathrm{s}} + G_{\mathrm{c2}}\right) + S_1 \cdot G_{\mathrm{c1}} - \left(S_1 + S_2\right)U_{\mathrm{e}} \tag{S24}$$

where S_1 and S_2 are the corresponding areas of S1 and S2, respectively, G_{c2} is the energy release rate of interfacial fracture in S2 area, G_{c1} is the film-substrate re-adhesion energy induced by the water nanolayer in S1 area, and U_{e} is the elastic strain energy stored in two areas due to the residual stress. Take $G_{\mathrm{c2}} = 0.1$ J m^{-2}, $G_{\mathrm{c1}} = 0.01$ J m^{-2}, $U_{\mathrm{e}} = 0.223$ J m^{-2}, the elastic modulus $E_{\mathrm{f}} = 29$ GPa, the Poisson's ratio $v = 0.27$, the same parameters as those in our previous work on web buckles[15]. The above equations (including Eqs. S(15), S(19)-S(24)) are numerically solved. The total energies for different sectional profiles (Fig. 2g) as a function of the propagation position are plotted in Fig. 4e.

15. Prediction of the equilibrium positions by our theoretical model

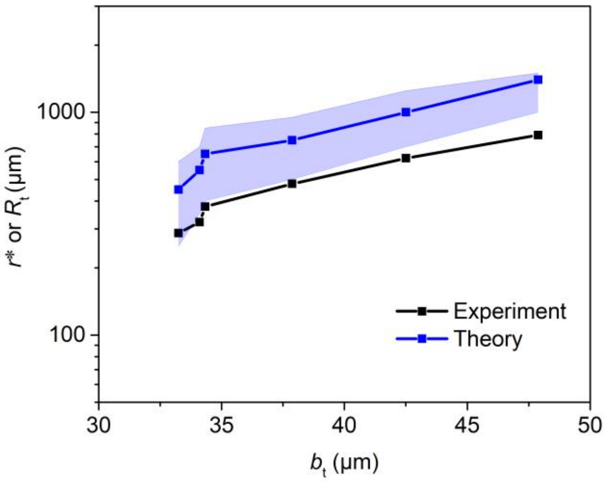

Supplementary Fig. 14 | Comparison between the curvature radii (*r) predicted by our theoretical model and the radii (R_{t}) from our experimental measurements for the arc buckles with different half widths (b_{t}).** The colored region indicates the possible range of radii where the net energy change is calculated to be negative, meaning the possible positions of buckles. Source data are provided as a Source Data file.

Supplementary references

1. Liu, L. et al. Phase-selective synthesis of 1T' MoS_2 monolayers and heterophase bilayers. *Nat. Mater.* **17**, 1108-1114 (2018).
2. Yang, H. et al. Highly scalable synthesis of MoS_2 thin films with precise thickness control via polymer-assisted deposition. *Chem. Mater.* **29**, 5772-5776 (2017).
3. Hui, Y. Y. et al. Exceptional tunability of band energy in a compressively strained trilayer MoS_2 sheet. *Acs Nano*. **7**, 7126-7131 (2013).
4. Hutchinson, J. W. & Suo, Z. Mixed mode cracking in layered materials. *Adv. Appl. Mech*. **29**, 63-191 (1991).
5. Hay, J., Agee, P. & Herbert, E. Continuous stiffness measurement during instrumented indentation testing. *Exp. Techniques*. **34**, 86-94 (2010).
6. Hay, J. & Crawford, B. Measuring substrate-independent modulus of thin films. *J. Mater. Res.* **26**, 727-738 (2011).
7. Owens, D. K. & Wendt, R. C. Estimation of the surface free energy of polymers. *J. Appl. Polym. Sci.* **13**, 1741-1747 (1969).
8. Annamalai, M. et al. Surface energy and wettability of van der Waals structures. Nanoscale. **8**, 5764-5770 (2016).
9. Kresse, G. & Furthmüller, J. Efficiency of ab-initio total energy calculations for metals and semiconductors using a plane-wave basis set. *Comp. Mater. Sci.* **6**, 15-50 (1996).
10. Blöchl, P. E. Projector augmented-wave method. *Phys. Rev. B*. **50**, 17953 (1994).
11. Perdew, J. P., Burke, K. & Ernzerhof, M. Generalized gradient approximation made simple. *Phys. Rev. Lett.* **77**, 3865 (1996).
12. Grimme, S., Antony, J., Ehrlich, S. & Krieg, H. A consistent and accurate ab initio parametrization of density functional dispersion correction (DFT-D) for the 94 elements H-Pu. *J. Chem. Phys*. **132**, 154104 (2010).
13. Xiang, Y. et al. Monolayer MoS_2 on sapphire: an azimuthal reflection high-energy electron diffraction perspective. *2D Mater*. **8**, 025003 (2020).
14. Chelli, R., Procacci, P., Righini, R. & Califano, S. Critical Review: Adhesion in surface micromechanical structures. *J. Chem. Phys.* **111**, 8569-8575 (1999).
15. Ren, H. et al. Watching Dynamic Self-Assembly of Web Buckles in Strained MoS_2 Thin Films. *Acs Nano.* **13**, 3106-3116 (2019).
