# Supplementary material for: Water nanolayer facilitated solitary-wave-like blisters in MoS2 thin films
Source: Nat Commun. 2023 Jul 19;14:4324. doi: 10.1038/s41467-023-40020-7 (PMC10356837; doi:10.1038/s41467-023-40020-7)
Supplement: Supplementary file 3 — Description of Additional Supplementary Files [file 41467_2023_40020_MOESM3_ESM.pdf]

Description of Additional Supplementary Files

Supplementary Movie 1: Dynamic propagation of SWLB and web buckles

Supplementary Movie 2: Propagation of a single SWLB
